# The complete plastomes of seven *Peucedanum* plants: comparative and phylogenetic analyses for the *Peucedanum* genus

**DOI:** 10.1186/s12870-022-03488-x

**Published:** 2022-03-07

**Authors:** Chang-Kun Liu, Jia-Qing Lei, Qiu-Ping Jiang, Song-Dong Zhou, Xing-Jin He

**Affiliations:** Key Laboratory of Bio-Resources and Eco-Environment of Ministry of Education, College of Life Sciences, Sichuan University, Chengdu, 610065 China

**Keywords:** Apiaceae, *Peucedanum*, Plastome evolution, Phylogenomics, DNA barcoding

## Abstract

**Background:**

The *Peucedanum* genus is the backbone member of Apiaceae, with many economically and medically important plants. Although the previous studies on *Peucedanum* provide us with a good research basis, there are still unclear phylogenetic relationships and many taxonomic problems in *Peucedanum*, and a robust phylogenetic framework of this genus still has not been obtained, which severely hampers the improvement and revision of taxonomic system for this genus. The plastid genomes possessing more variable characters have potential for reconstructing a robust phylogeny in plants.

**Results:**

In the current study, we newly sequenced and assembled seven *Peucedanum* plastid genomes. Together with five previously published plastid genomes of *Peucedanum*, we performed a comprehensively comparative analyses for this genus. Twelve *Peucedanum* plastomes were similar in terms of genome structure, codon bias, RNA editing sites, and SSRs, but varied in genome size, gene content and arrangement, and border of SC/IR. Fifteen mutation hotspot regions were identified among plastid genomes that can serve as candidate DNA barcodes for species identification in *Peucedanum*. Our phylogenetic analyses based on plastid genomes generated a phylogeny with high supports and resolutions for *Peucedanum* that robustly supported the non-monophyly of genus *Peucedanum*.

**Conclusion:**

The plastid genomes of *Peucedanum* showed both conservation and diversity. The plastid genome data were efficient and powerful for improving the supports and resolutions of phylogeny for the complex *Peucedanum* genus. In summary, our study provides new sights into the plastid genome evolution, taxonomy, and phylogeny for *Peucedanum* species.

**Supplementary Information:**

The online version contains supplementary material available at 10.1186/s12870-022-03488-x.

## Background

*Peucedanum* L. is one of the largest genera of Apiaceae [1–3], which was once placed in the tribe Peucedaneae [1, 4, 5], but now in the tribe Selineae [2]. The genus comprises 100-120 species worldwide that are widely distributed in Eurasia and South Africa (and sometimes Australia) [2, 3, 6], with Europe and East Asia as distribution centers [7]. Of those, forty species are distributed in China with 33 of them endemic [3].

The genus *Peucedanum* is taxonomically notorious within Apiaceae family, especially described as “*Peucedanum* problem” by Downie et al. [8]. Its members are characterized by dorsally compressed mericarps with slightly prominent dorsal ribs, narrowly winged lateral ribs, as well as a broad commissure [2, 3]. However, the genus is extremely heterogenous and exhibits great diversity in life-forms, leaf and fruit structures, and chemical constituents [9]. Hence, several researchers are prone to divide this genus into smaller and presumably more natural units. For example, Pimenov and Leonov [5] suggested that all members of *Peucedanum* except 8-10 species included in sect. *Peucedanum* should be transferred to other genera. Based on morphological and phytochemical evidences, Reduron et al. [10] separated the genera *Cervaria* Wolf, *Imperatoria* L., *Oreoselinum* Mill., *Pteroselinum* Rchb., *Thysselinum* Adans., *Xanthoselinum* Schur and *Holandrea* Reduron from *Peucedanum*. Winter et al. [11] established three new genera (*Afrosciadium* P.J.D. Winter, *Nanobubon* Magee and *Notobubon* B.-E. van Wyk) to accommodate the African peucedanoid species and transferred 24 *Peucedanum* species into *Afroligusticum* C. Norman, *Cynorhiza* Eckl. & Zeyh., and *Lefebvrea* A. Rich. However, due to the varied morphological features of leaf division, bracteoles, and mericarps, distinguishing separate genera from *Peucedanum* is extremely difficult [2, 3]. Therefore, the generic limits of *Peucedanum* based on morphological characters faces challenges.

A robust phylogenetic framework could provide a valuable information to aid the generic delimitation of *Peucedanum*. Previously, a few molecular phylogenies of *Peucedanum* based on single or multiple-locus DNA sequence data, such as nuclear ribosomal DNA internal transcribed spacer (ITS), plastid DNA *rpl*16 and *rps*16 intron, have been performed, yet these analyses failed to recognize *Peucedanum* as a monophyletic group [2, 12–16]. This phenomenon infers that re-evaluating the generic limits of *Peucedanum* may be essential. Nevertheless, weak supports and low resolutions of these phylogenetic trees could not provide sufficient information to support the improvement of taxonomy for *Peucedanum*. Therefore, additional molecular data are urgent to reconstruct a strong phylogeny.

In addition, several species of *Peucedanum* are highly appreciated as traditional medicinal herbs due to their versatile therapeutic properties [17]. Among them, *Peucedanum praeruptorum* Dunn, known as “Baihu Qianhu”, is an excellent representation. The dried root of *P. praeruptorum* has been utilized as traditional Chinese medicine for more than 1500 years, which is generally used to treat respiratory diseases, pulmonary hypertension, chest pain, as well as symptomatic coughs and dyspnea [18]. However, most *Peucedanum* species exhibit abundant intraspecific variations in morphology that make it difficult to accurately identify species. In order to assure medicinal quality, it is, therefore, necessary to develop specific DNA marker for *Peucedanum* species authentication.

The plastid genome (plastome) is one of the three DNA genomes (with nuclear and mitochondrial genomes) in plants. The genome is uniparentally inherited, lacks recombination, and possesses highly variable characters in flowering plants; hence, it has the potential to significantly improve the supports and resolutions of the phylogeny [19–22]. Furthermore, a typical plastome comprises two inverted repeats regions (IRs) of 22-25 kb separated by the large single copy region (LSC) of 82-90 kb and small single copy region (SSC) of 15-20 kb and generally encodes 110-130 unique genes [23, 24]. Comparative analysis of plastome could reveal the diversity of plastome in structural organization, gene arrangement and content that deepens our understanding of adaptive evolution for plant lineages and identify suitable mutation hotspots for species authentication [21, 25, 26]. Hence, with the development of next-generation sequencing and bioinformatics technologies, plastomes have been extensively and successfully used for plant phylogenetic analyses and development of specific DNA barcodes in recent years [25–32].

Currently, although six plastomes of *Peucedanum* species were submitted in GenBank [33–36], the plastid phylogenomic analysis of the genus has not been conducted. In this study, we newly sequenced the plastomes of seven *Peucedanum* taxa. In conjunction with the previously reported five plastomes of *Peucedanum*, we carried out a comprehensive analysis of plastomes for this taxonomically difficult plant group. Our aims were to: (1) investigate the plastome features of *Peucedanum* plants; (2) screen out suitable mutation hotspot regions from plastome as candidate DNA barcodes for species identification of *Peucedanum*; (3) test the power of plastome for improving the supports and resolutions of phylogeny in the complex *Peucedanum* genus. Overall, our results will well lay the foundation for the phylogenetic and taxonomic studies of *Peucedanum*.

## Results

### Plastome features of *Peucedanum*

Illumina sequencing generated 36,875,778-44,140,972 paired-end clean reads for the seven *Peucedanum* samples. Among them, 712,889 to 6,125,929 reads were mapped to the final assembly. Based on these data, we obtained seven high-quality *Peucedanum* plastomes, with coverage ranging from 730.073× to 6,266.178× (Table S1).

**Fig. 1:**
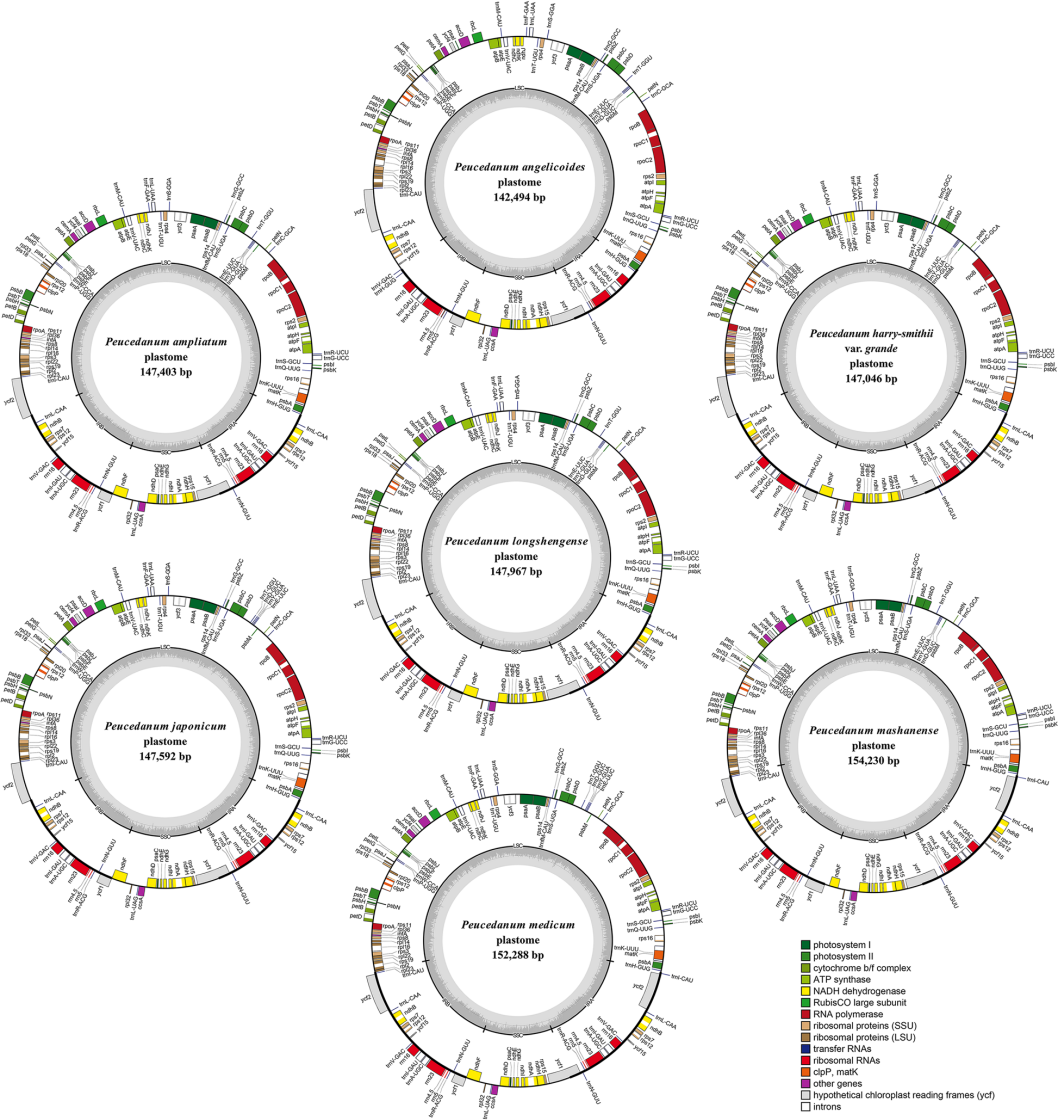
Maps of seven *Peucedanum* plastomes. Genes shown outside of the outer layer circle are transcribed clockwise, while those insides are transcribed counterclockwise. The genes belonging to different functional groups are color-coded. The dark gray area of the inner circle denotes the GC content of plastome

Overall size of plastomes ranged from 142,494 bp (*P. angelicoides* Wolff ex Kretschm.) to 156,899 bp (*P. insolens* Kitag.) for the twelve *Peucedanum* samples (Table 1). All of them shown typically quadripartite structure, including a pair of inverted repeats regions (IRs, 12,594-27,495 bp), a large single copy region (LSC, 84,492-99,934 bp), and a small single copy region (SSC, 16,665-17,627 bp) (Fig. 1, Table 1). The total GC content of the twelve plastomes ranged from 37.4% to 37.7% (Table 1). The twelve plastomes encoded 113-114 unique genes, including 79-80 protein-coding genes, 29-30 tRNA genes, and four rRNA genes (Table 1, Table S2). The *ycf*15 gene was lost in *P. delavayi* Franch*.* and *P. insolens*; the *trn*T-GGU gene was absent in *P. praeruptorum* and *P. harry-smithii* var. *grande* (K.T.Fu) Shan et Sheh (Table S2).

**Table 1:** Comparison of plastome features among *Peucedaum* plants

Taxon	Total length (bp)	LSC (bp)	SSC (bp)	IR (bp)	Total GC content (%)	Total genes (unique)	Protein coding genes (unique)	rRNA genes (unique)	tRNA genes (unique)
*P. ampliatum*	147,403	92,526	17,519	18,679	37.6	114	80	4	30
*P. angelicoides*	142,494	99,934	17,372	12,594	37.4	114	80	4	30
*P. chujaense*	147,839	93,335	17,590	18,457	37.4	114	80	4	30
*P. delavayi*	155,552	85,276	17,394	26,441	37.6	113	79	4	30
*P. harry-smithii* var*. grande*	147,046	92,135	17,627	18,642	37.6	113	80	4	29
*P. insolens*	156,899	84,492	17,417	27,495	37.7	113	79	4	30
*P. japonicum*	147,592	92,804	17,576	18,606	37.5	114	80	4	30
*P. longshengense*	147,967	93,265	17,572	18,565	37.5	114	80	4	30
*P. mashanense*	154,230	86,957	16,665	25,304	37.4	114	80	4	30
*P. medicum*	152,288	86,645	17,571	24,036	37.5	114	80	4	30
*P. praeruptorum*	147,197	92,161	17,610	18,713	37.6	113	80	4	29
*P. terebinthaceum*	147,925	93,368	17,571	18,493	37.5	114	80	4	30

**Fig. 2:**
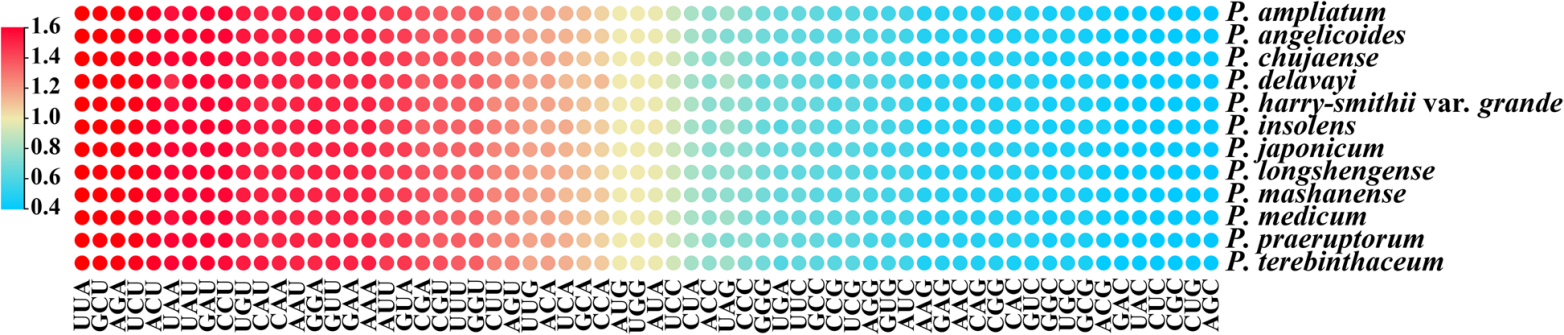
The RSCU values of all concatenated protein-coding genes for 12 *Peucedanum* plastomes. Color key: the red values represent higher RSCU values while the blue values indicate lower RSCU values

In order to analyze the codon usage of *Peucedanum* plastomes, 79 protein-coding genes were extracted and connected for each plastome. These sequences were 66,552-68,130 bp in length and encoded 22,184-22,710 codons. The Leu was encoded by the highest number of codons (2,347-2,404), while the Cys was the least (234-243) in all plastomes (Table S3). In addition, relative synonymous codon usage (RSCU) values of all codons ranged from 0.32 to 2.01 in the twelve plastomes (Table S3). Specifically, RSCU values of 30 codons were greater than 1.00 in all plastomes, whereas the codon AUA with RSCU > 1.00 was only detected in *P. insolens* plastomes (Fig. 2). All codons with RSCU > 1.00 were ended with A/U, except UUG (Fig. 2).

The potential RNA editing sites for 35 protein-coding genes of the twelve plastomes were detected. A total of 56-60 potential RNA editing sites were identified (Table S4, Fig. S1). All detected RNA editing sites were Cytosine to Uracil (C-U) conversion and most of them occurred in the second codon position (42-45), followed by the first codon position (12-16), but no sites situated in the third codon position (Fig. S1A). Moreover, the *ndh*B gene contained the highest number of RNA editing sites ranging from 10 to 11 (Fig. S1B).

**Fig. 3:**
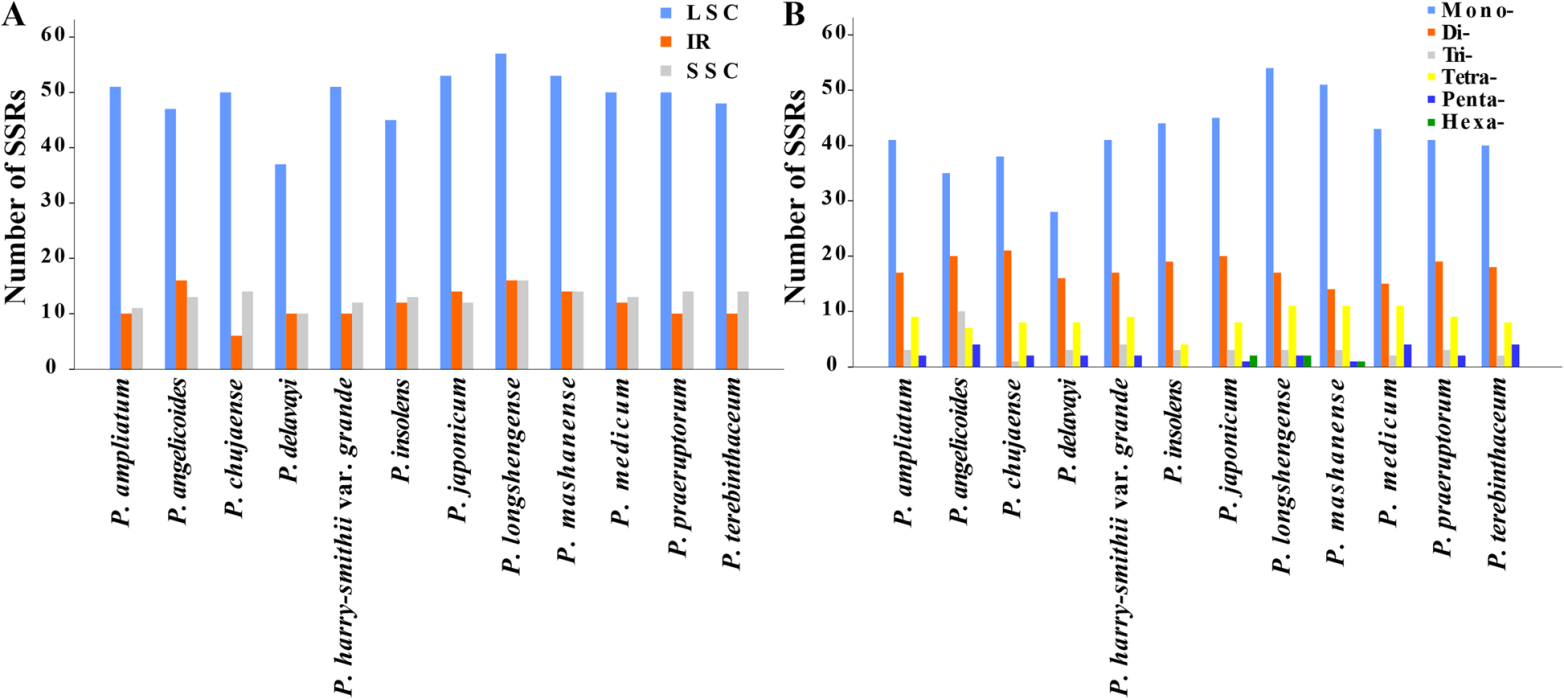
Analyses of simple sequence repeats (SSRs) in twelve *Peucedanum* plastomes: **A** presence of SSRs in LSC, SSC, and IR; **B** numbers of different repeat types

The total number of SSRs ranged from 58 to 89 among the twelve *Peucedanum* plastomes (Fig. 3, Table S5). Most of the SSRs distributed in the LSC region for all plastomes (Fig. 3A). Among these SSRs, the mononucleotide repeats were the most abundant (28-54), followed by the dinucleotides (14-21) (Fig. 3B). In addition, bases A and T were the dominant elements for all identified SSRs in the twelve plastomes.

### Plastome comparison and hotspots identification

**Fig. 4:**
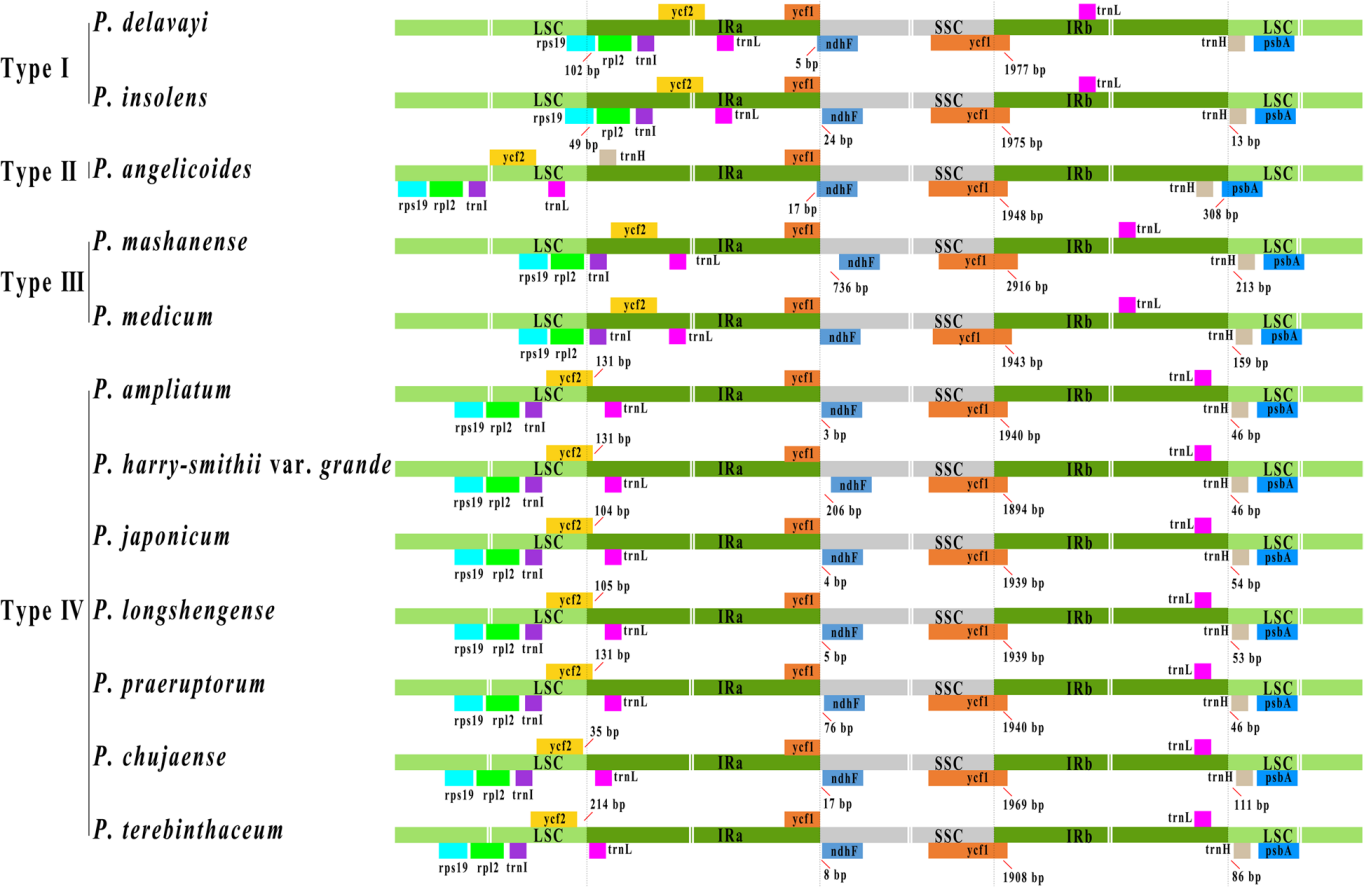
Comparison of the borders of the LSC, SSC, and IR regions among twelve *Peucedanum* plastomes

The borders of IRa/SSC, IRb/SSC, and IRb/LSC among the twelve *Peucedanum* plastomes were slightly conserved: the IRa/SSC junctions of most samples were located between *ycf*1 gene and *ndh*F gene, but expanded into *ndh*F gene in *P. delavayi* and *P. angelicoides*; the boundaries of IRb/SSC fell into *ycf*1 gene; the IRb/LSC borders of most samples were located between genes of *trn*L and *trn*H, but extremely expanded into *psb*A gene in *P. angelicoides* (Fig. 4). However, the junctions of IRa/LSC of plastomes within *Peucedanum* genus were divergent and could be classified into four different types. The junctions of IRa/LSC fell into the *rps*19 gene in *P. delavayi* and *P. insolens*, belonging to the type I; the IRa/LSC borders contracted to the intergenic region of *trn*L-*trn*H in *P. angelicoides* (type II) while moved to the intergenic regions of *rpl*2-*trn*I in *P. mashanense* Shan et Sheh and *P. medicum* Dunn (type III); the IRa/LSC borders of most remainder *Peucedanum* plants fell into the *ycf*2 gene, but contracted to the intergenic regions of *ycf*2-*trn*L in *P. chujaense* K. Kim, S.H. Oh, C.S. Kim & C.W. Park and *P. terebinthaceum* (Fisch.) Fisch. ex Turcz. (type IV) (Fig. 4).

**Fig. 5:**
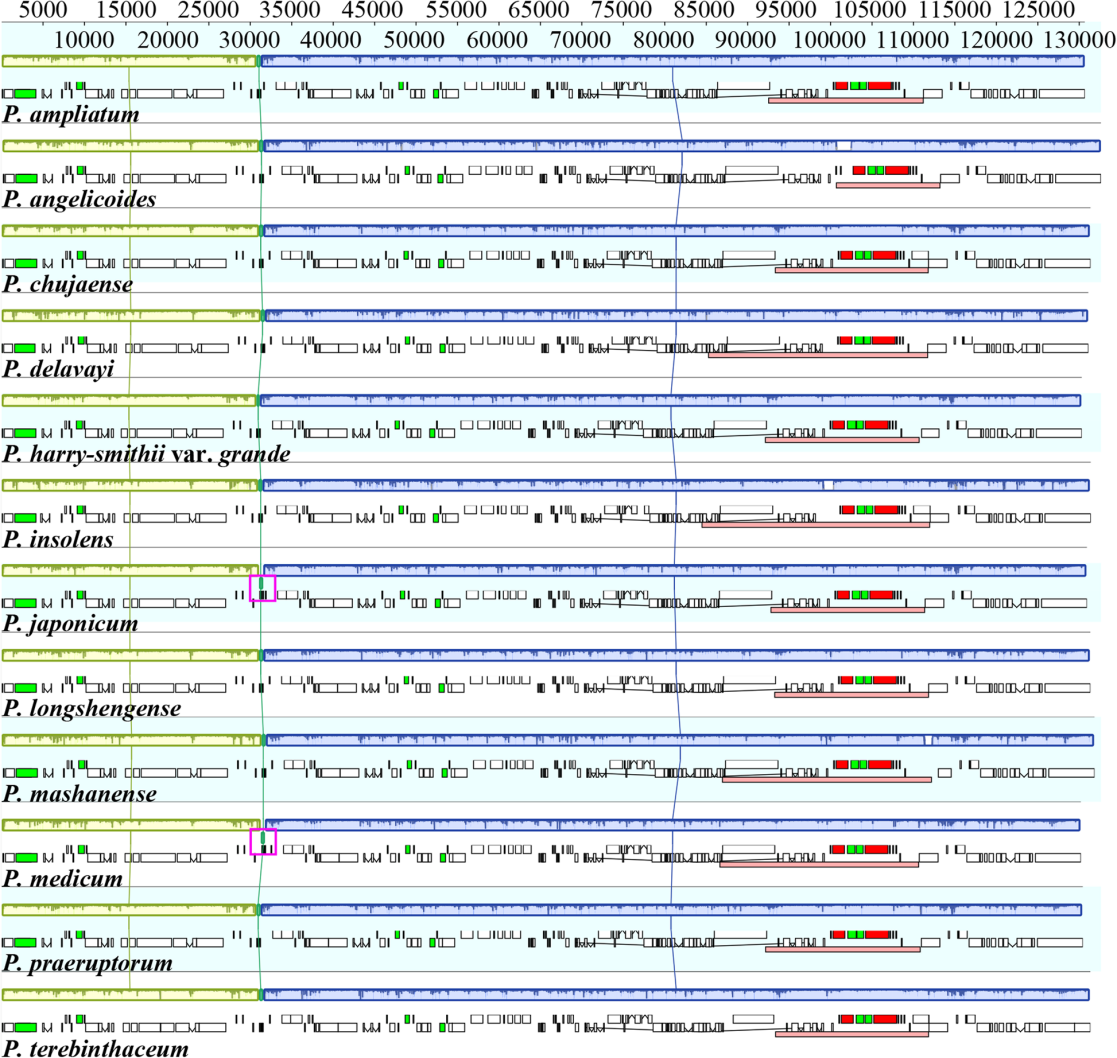
Mauve alignment of twelve *Peucedanum* plastomes. Local collinear blocks within each alignment are represented by blocks of the same color connected with lines. The colored boxes are the inversion of the *trn*Y-*trn*D-*trn*E gene

**Fig. 6:**
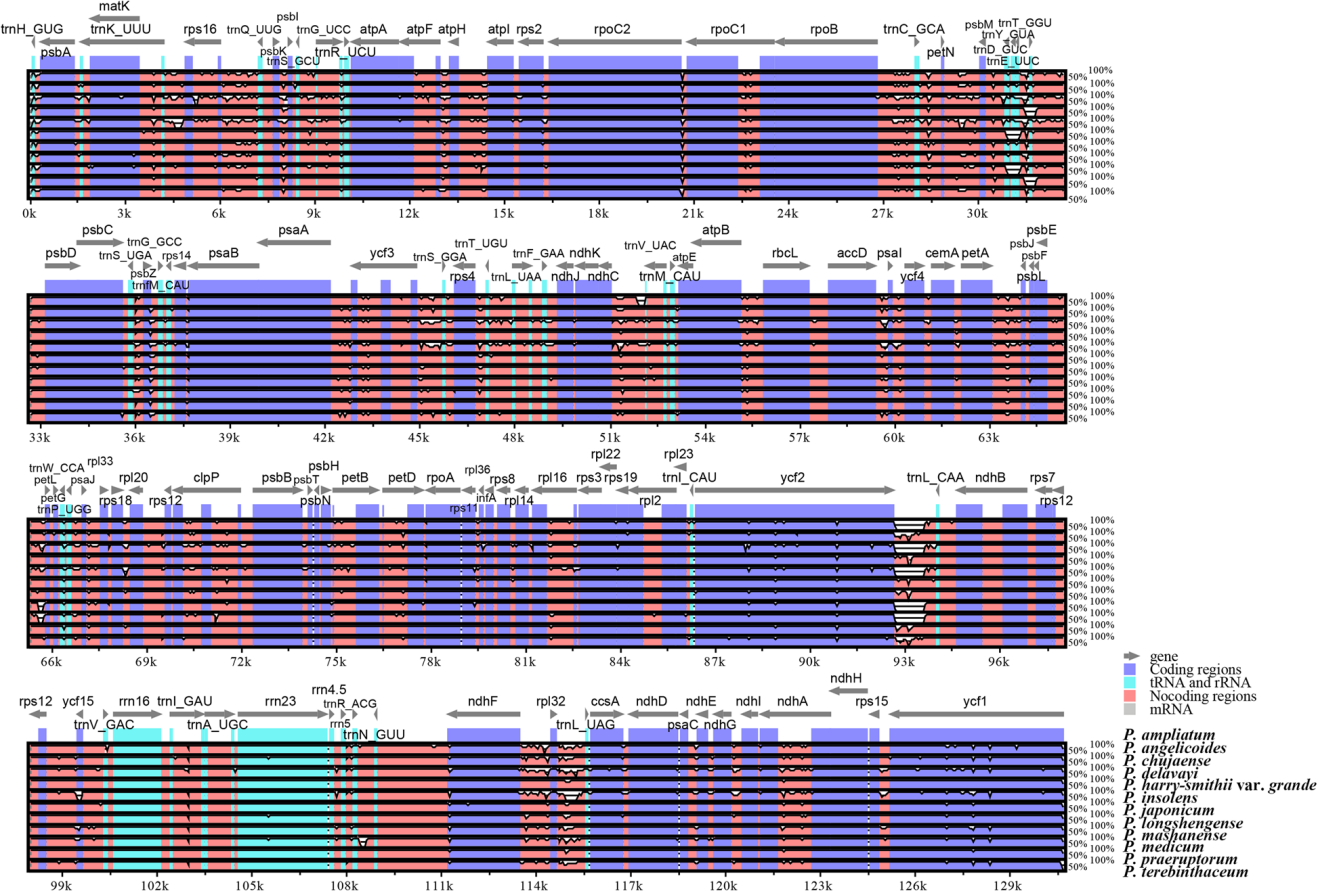
Sequence identity plots for the twelve *Peucedanum* plastomes

**Fig. 7:**
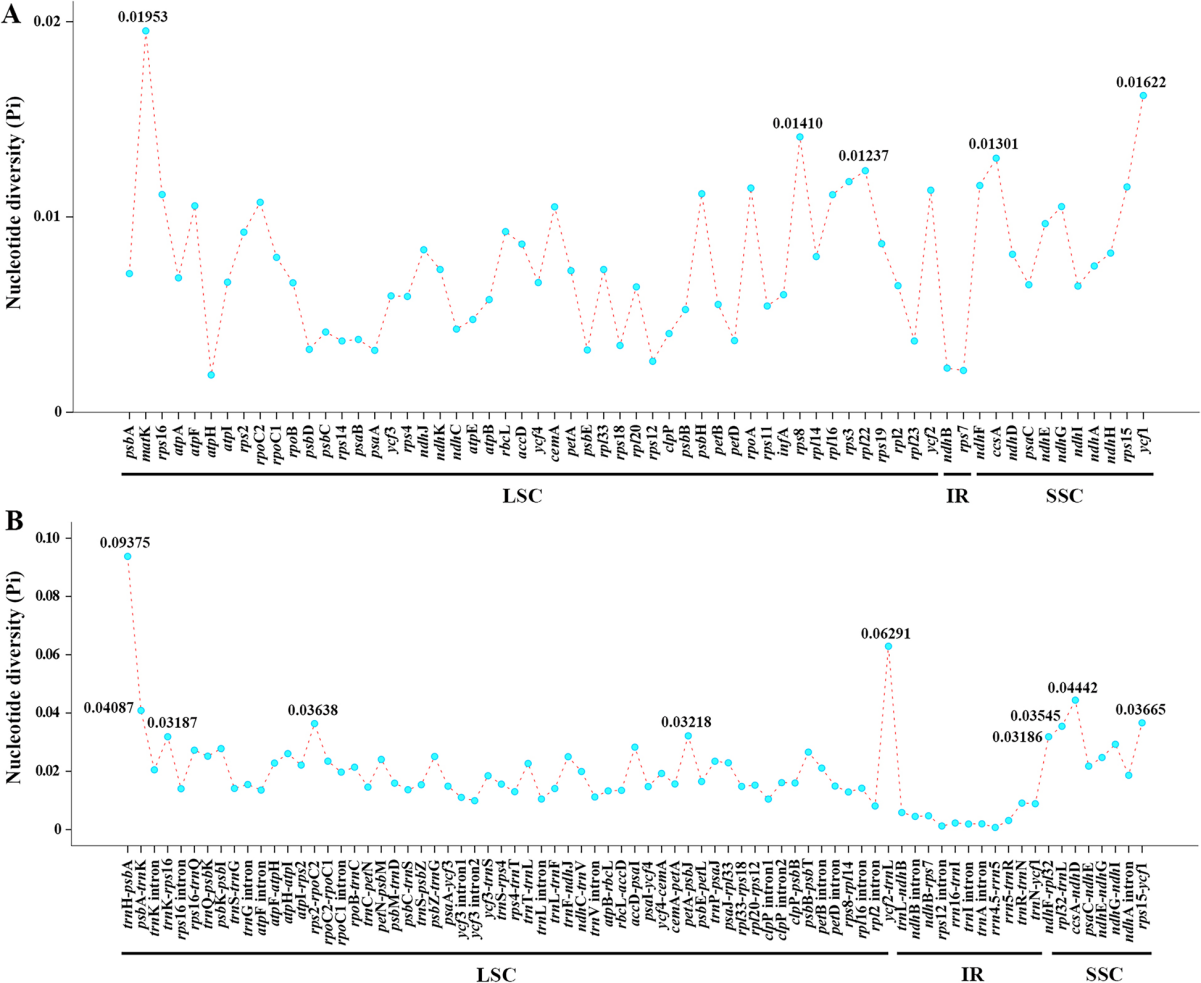
Comparative analysis of the nucleotide diversity (Pi) values among the twelve *Peucedanum* plastomes: **A** protein coding genes; **B** non-coding and intron regions

The genes arrangement of the twelve *Peucedanum* plastomes was relatively conserved, except for an inversion of the *trn*Y-*trn*D-*trn*E gene detected in *P. japonicum* Thunb. and *P. medicum* (Fig. 5). However, the whole plastome sequences shared low similarity among the twelve *Peucedanum* samples, identifying 7,350 variation sites in the 142,197 alignment positions (Fig. 6). According to the sequence divergences, the 15 mutation hotspot regions were selected as candidate DNA barcodes, including five protein coding genes–*ccs*A, *mat*K, *rpl*22, *rps*8, *ycf*1–which showed the Pi > 0.01200 (Fig. 7A) and 10 non-coding regions–*ccs*A-*ndh*D, *ndh*F-*rpl*32, *pet*A-*psb*J, *psb*A-*trn*K, *rpl*32-*trn*L, *rps*15-*ycf*1, *rps*2-*rpoC*2, *trn*H-*psb*A, *trn*K-*rps*16, *ycf*2-*trn*L–which showed the Pi >0.03100 (Fig. 7B).

### Phylogenetic analyses

**Fig. 8:**
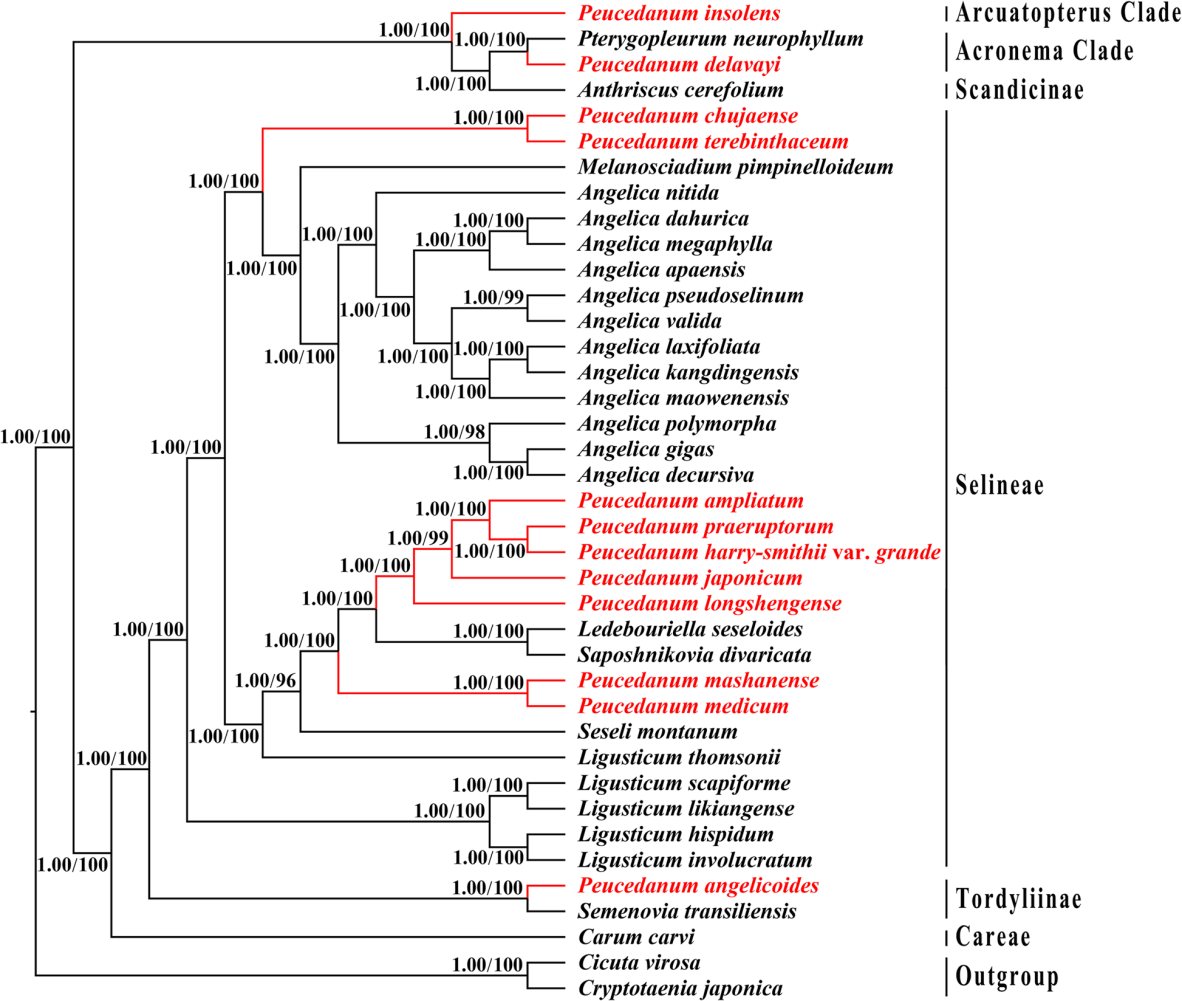
Phylogeny of the 39 taxa inferred from Maximum likelihood (ML) and Bayesian inference (BI) analyses. Numbers represent Bayesian posterior probabilities (PP) and maximum likelihood bootstrap values (BS)

The analyses of ML and BI generated the identical tree topology. The Fig. 8 illustrated the phylogeny, including two types of support values: BI posterior probabilities (PP) and ML bootstrap values (BS). Both analyses robustly supported that members of *Peucedanum* not clustered as monophyletic but fell into four clades: (1) *P. insolens* was placed in Arcuatopterus clade (PP = 1.00, BS = 100); (2) *P. delavayi* was sister to *Pterygopleurum neurophyllum* (Maxim.) Kitag., belonging to Acronema clade (PP = 1.00, BS = 100); (3) *P. angelicoides* clustered with *Semenovia transiliensis* Regel & Herder constituting Tordyliinae (PP = 1.00, BS = 100); (4) the remainders were included in Selineae (PP = 1.00, BS = 100). Most of the *Peucedanum* accessions fell into the tribe Selineae, while these samples were also not clustered in a clade. Within Selineae, three major lineages for *Peucedanum* accessions were recognized: *P. chujaense* and *P. terebinthaceum* formed a clade that was relatively distant from others (PP = 1.00, BS = 100); *P. mashanense* was clustered with *P. medicum* (PP = 1.00, BS = 100); *P. ampliatum* K.T. Fu, *P. praeruptorum*, *P. harry-smithii* var. *grande*, *P. japonicum*, and *P. longshengense* Shan et Sheh formed a clade (PP = 1.00, BS = 100), in which *P. longshengense* firstly diverged from the remainders (PP = 1.00, BS = 100), followed by *P. japonicum* (PP = 1.00, BS = 99), and the sub-clade *P. praeruptorum* + *P. harry-smithii* var. *grande* sister to *P. ampliatum* (PP = 1.00, BS = 100). In addition, the phylogenetic relationships among non-*Peucedanum* species inferred in this study were generally consistent with the previous work [37], but our results gave the higher support values for these relationships, showing PP = 1.00 and BS ≥ 96 for all nodes.

## Discussion

### Comparison of the plastomes in *Peucedanum*

In this study, we sequenced and assembled seven plastomes of *Peucedanum* and performed a comprehensive comparative analyses of these plastomes with five other published plastomes of this genus obtained from GenBank. All *Peucedanum* plastomes showed a typically quadripartite structure, including a pair of inverted repeats regions separated by the large single copy region and small single copy region [33–36]. In addition, codon bias, RNA editing sites, and the distribution and constituent of SSRs were quite similar among twelve *Peucedanum* plastomes. These results suggested that *Peucedanum* plastome is conserved in terms of genome structure, codon bias, RNA editing sites, and SSRs. It is worth noting that this phenomenon is commonly found in other genera of flowering plants [38–40], which may be related to maintaining the stability of plastome function.

However, we also detected obvious diversity among the twelve *Peucedanum* plastomes. First, the overall sizes of plastomes varied from 142,494 bp (*P. angelicoides*) to 156,899 bp (*P. insolens*) among *Peucedanum* plants. Second, the *ycf*15 gene was lost in *P. delavayi* and *P. insolens*, whereas the *trn*T-GGU gene was absent in *P. praeruptorum* and *P. harry-smithii* var. *grande*. The loss of the *ycf*15 gene has been detected in a wide diversity of lineages in the angiosperms [41–44], which may occur independently during the evolution of these lineages, hence, it may not provide relevant phylogenetic information. However, the loss of *trn*T-GGU gene was only observed in *P. praeruptorum* and *P. harry-smithii* var. *grande* and not identified in other members of Apiaceae [26, 37, 39], and thus it can be used as specific molecular marker to recognize this group. Third, the inversion of the *trn*Y-*trn*D-*trn*E gene was detected in *P. japonicum* and *P. medicum*, which has been observed in *Angelica* L. species [26]. Finally, we observed extensive expansion and contraction of the IR regions among *Peucedanum* samples, recognizing four types of SC/IR border. All patterns have been observed in other genera of Apiaceae [26, 37, 39]. Overall, these plastome divergences detected among *Peucedanum* members further implied the non-monophyly of the *Peucedanum* genus.

### Phylogeny inference

The utilization of a small number of DNA fragments for phylogenetic analysis may cause phylogenetic errors and thus result in the incongruent topology among different DNA sequences [45–47]. Hence, using few DNA sequences to infer the phylogeny of plant species might be frequently insufficient and inappropriate, especially at low taxonomic levels [26, 47]. The plastome sequence possesses highly variable characters and thus has the tremendous potential power to reconstruct the robust phylogeny at low taxonomic levels [19–22, 31]. Therefore, we performed plastid phylogenomic analyses for *Peucedanum* genus in this study. As expected, compared to previous phylogenetic studies by using single or multiple locus DNA sequences [2, 12–16], our phylogenetic analyses based on whole plastome sequences generated a robust phylogenetic framework for *Peucedanum* members, all nodes showing PP = 1.00 and BS ≥ 96. This result justifies that the plastome sequence is powerful and effective to improve the supports and resolutions of phylogeny for *Peucedanum* genus.

The *Peucedanum* genus was not recovered as monophyletic in our phylogenomic analyses, which was congruent with the previous studies that used ITS data and two plastid DNA regions (*rpl*16 and *rps*16 intron) [2, 12–16]. It is further supported by the great divergence of leaf epidermal morphologies [48], and fruit structures [49, 50] among *Peucedanum* members. These results justified that the *Peucedanum* genus is not a natural taxonomy unit. Therefore, the current taxonomy system of *Peucedanum* urgently needs to be improved and revised. Although the taxonomic treatment for *Peucedanum* members has not been performed in the current study due to the absence of the type species of *Peucedanum* (*P. officinale* L.), our results lay the foundations for the future taxonomic studies of *Peucedanum*.

The phylogenetic relationships among *P. japonicum*, *P. praeruptorum*, and *P. terebinthaceum* have long been controversial [14, 51, 52]. The phylogenetic analyses of Feng et al. [14] based on ITS sequences showed that *P. praeruptorum* was sister to *P. japonicum* that was relatively distant from *P. terebinthaceum*. However, the results of Ostroumova et al. [51] and Pimenov et al. [52] indicated that *P. praeruptorum* made a cluster with *P. terebinthaceum* being sister to *P. japonicum*. Our plastid phylogenomic analyses robustly supported that *P. japonicum* was sister to the clade consisting of *P. ampliatum*, *P. praeruptorum* and *P. harry-smithii* var. *grande*, in which the subclade of *P. praeruptorum* + *P. harry-smithii* var. *grande* diverged from *P. ampliatum*; *P. terebinthaceum* and *P. chujaense* clustered into a clade that was distant from all other *Peucedanum* members. The relationships recovered in the current study are different from those of previous studies [14, 51, 52]. With high supports and resolutions, our plastid-based phylogenetic analyses provide new sights into the inter-species relationship within *Peucedanum*.

### Potential DNA barcodes

The accurate species identification has always been a serious challenge faced by taxonomists. The advent of DNA barcoding technology, which uses the short DNA sequences with sufficient variations to discriminate species [53], promises to resolve this difficulty. The mitochondrial gene cytochrome oxidase 1 has been proven to be effective and reliable as a standard DNA barcode for animal species identification [54–57]. However, in plants, reliable species identification based on universal DNA barcodes, i.e., *rbc*L, *mat*K, *trn*H-*psb*A, is frequently problematic [58–62]. As expected, we found that the variation in *rbc*L gene was relatively low (Pi = 0.00925) among *Peucedanum* plants. Hence, this region may have limited power to discriminate *Peucedanum* species.

Based on sequence variations, five protein coding genes (*ccs*A, *mat*K, *rpl*22, *rps*8, *ycf*1) and ten non-coding regions (*ccs*A-*ndh*D, *ndh*F-*rpl*32, *pet*A-*psb*J, *psb*A-*trn*K, *rpl*32-*trn*L, *rps*15-*ycf*1, *rps*2-*rpoC*2, *trn*H-*psb*A, *trn*K-*rps*16, *ycf*2-*trn*L) were selected, which were potentially useful for species identification in *Peucedanum* genus. Among them, *mat*K gene and *trn*H-*psb*A region are members of universal DNA barcodes [62]; *ccs*A, *rpl*22, *ycf*1, *ccs*A-*ndh*D, *ndh*F-*rpl*32, *trn*K-*rps*16, and *ycf*2-*trn*L have been chosen as promising DNA barcodes in other plants [26, 39, 63–65]; and *pet*A-*psb*J, *rpl*32-*trn*L, and *rps*15-*ycf*1 regions have been widely used for phylogenetic analyses [66–70]. In a future study, we will test whether or not these sequences can serve as reliable DNA barcodes for species identification within *Peucedanum* genus.

## Conclusion

This study is the first attempt to comprehensively investigate the plastome features and infer phylogeny by using plastome data for *Peucedanum* genus. Comparative analyses found that plastomes of *Peucedanum* are conserved in terms of genome structure, codon bias, RNA editing sites, and SSRs, but varied in genome size, gene content and arrangement, and border of SC/IR. The plastid phylogenomic analyses prove that plastome data are efficient and powerful for improving the supports and resolutions of *Peucedanum* phylogeny and robustly support that *Peucedanum* is not a monophyletic group. In addition, fifteen mutation hotspot regions are identified across the plastomes that can serve as potential DNA barcodes for species identification in *Peucedanum*. Overall, our study lays the foundations for the future phylogeny and taxonomy of *Peucedanum*.

## Methods

### Plant material, DNA extraction, plastome sequencing and assembly

The fresh young leaves of seven *Peucedanum* taxa were collected from the wild and the greenhouse in College of Life Sciences, Sichuan University, and then dried with silica gel. The formal identifications of all samples were undertaken by Professor Xingjin He (Sichuan University). The voucher specimens were deposited at the herbarium of Sichuan University (Chengdu, China) under deposition numbers of LCK2020001- LCK2020004, LZL2020085, JQP19082303, and JQP19082505 (Table S6). Total DNA was extracted from ~20 mg silica-gel-dried leaves with the CTAB method [71]. Genomic DNA then was fragmented into 400 bp to construct the pair-end library, following the manufacturer's protocol (Illumina, San Diego, CA, USA). The libraries were sequenced on the Illumina NovaSeq platform at Personalbio (Shanghai, China). Raw data were filtered using fastP v0.15.0 (-n 10 and -q 15) to obtain high quality reads [72]. Then high-quality reads were used to assemble the whole plastome with NOVOPlasty v2.6.2 [73], with the default parameters and *rbc*L sequence from *P. japonicum* (JF943288) as seed.

### Genomic annotation and feature analyses

The assembled plastomes were annotated using web server CPGAVAS2 (http://www.herbalgenomics.org/cpgavas2) [74]. The start and stop codons and intron positions were manually corrected according to plastomes of congeneric species in Geneious v9.0.2 [75]. The maps of annotated plastomes were drawn using the online program OrganellarGenomeDRAW (OGDRAW) [76].

Five whole plastomes of *Peucedanum* (*P. chujaense*, *P. delavayi*, *P. insolens*, *P. praeruptorum*, and *P. terebinthaceum*) were downloaded from NCBI. Together with newly sequenced plastomes, we investigated the codon usage of *Peucedanum* plastomes with the CodonW v1.4.2 program [77]. Then, we predicted the potential RNA editing sites of protein coding genes for the twelve *Peucedanum* plastomes by using the online program Predictive RNA Editor for Plants suite with a cutoff value of 0.8 [78]. Moreover, simple sequence repeats (SSRs) for each plastomes were detected with MISA (http://pgrc.ipk-gatersleben.de/misa/). The thresholds of repeat units were set as 10, 5, 4, 3, 3, and 3, for mono-, di-, tri-, tetra-, penta-, and hexanucleotides, respectively.

### Genomic comparison

We compared the boundaries of the LSC, SSC and IR regions among the twelve *Peucedanum* plastomes in Geneious v9.0.2 [75]. Then, the DNA rearrangements among *Peucedanum* plastomes were detected by using Mauve Alignment [79] implemented in Geneious v9.0.2 [75]. Furthermore, sequence divergence of *Peucedanum* plastomes was investigated using the mVISTA tool [80], with *P. ampliatum* set as the reference.

### Identification of divergence hotspots

In order to identify mutation hotspot regions, the protein coding genes, non-coding regions and intron regions of the twelve *Peucedanum* plastomes were extracted in Geneious v9.0.2 [75] and aligned with MAFFT v7.221 [81]. Then, alignments with more than 200 bp in length were used to evaluate nucleotide diversity (Pi) using DnaSP v5.0 [82]. The thresholds of Pi for protein coding gene and non-coding region were set as 0.01200 and 0.03100, respectively.

### Phylogenetic analyses

To infer the phylogenetic relationships among *Peucedanum* species, we reconstructed phylogenetic trees using 39 plastomes (Table S6, Table S7). *Cicuta virosa* L. and *Cryptotaenia japonica* Hassk. were chosen as outgroup to root the phylogenetic tree, according to the results of Wen et al. [37]. Sequence alignment was performed with the software MAFFT v7.221 [81], and adjusted and corrected manually when necessary. The unambiguous matrix was subjected to Maximum-Likelihood analyses (ML) and Bayesian Inference (BI). The ML phylogenetic tree was reconstructed in the program RAxML v8.2.8 [83] with 1000 replicates and GTRGAMMA model as the RAxML manual suggested. The BI analysis was performed by using MrBayes v3.2.7 [84] with the best-fit substitution model (TVM+I+G) determined by Modeltest v3.7 [85]. Two independent Markov chains were run for 1,000,000 generations, sampling every 100 generations. The first 25% of trees were discarded as burn-in and the remainder were used to generate the consensus tree. Results of phylogenetic analyses were visualized and edited in FigTree v1.4.2 [86].

## Supplementary Information

**Additional file 1: Fig. S1.** Analyses of RNA editing sites in twelve *Peucedanum* plastomes: (A) numbers of RNA editing sites distributed in different codon positions; (B) numbers of RNA editing sites presented in genes.

**Additional file 2: Table S1.** Summary of Illumina sequencing of *Peucedanum*.

**Additional file 3: Table S2.** List of unique genes identified in plastomes of *Peucedanum.*

**Additional file 4: Table S3.** Codon usage and relative synonymous codon usage (RSCU) values of protein-coding genes of the twelve *Peucedanum* plastomes.

**Additional file 5: Table S4.** RNA editing sites detected in the twelve plastome of *Peucedanum*.

**Additional file 6: Table S5.** Numbers of SSR motifs identified in the twelve *Peucedanum* plastomes.

**Additional file 7: Table S6.** Taxa newly sequenced in the present study with source, voucher and GenBank accession numbers.

**Additional file 8: Table S7.** Plastomes included in phylogenetic analyses with GenBank accession.

## Data Availability

The seven newly sequenced plastomes have been submitted into NCBI with accession numbers: OK336473-OK336479.

## Abbreviations

BI: Bayesian inference
bp: Base pair
BS: Bootstrap value
CTAB: Cetyl trimethylammonium bromide
IR: Inverted repeat
ITS: Internal transcribed spacer
LSC: Large single copy
ML: Maximum Likelihood
Pi: Nucleotide diversity
PP: Posterior probability
rRNA: Ribosomal RNA
RSCU: Relative synonymous codon usage
SSC: Small single copy
SSR: Simple sequence repeat
tRNA: Transfer RNA
