# Supplementary material for: The complete plastomes of seven Peucedanum plants: comparative and phylogenetic analyses for the Peucedanum genus
Source: BMC Plant Biol. 2022 Mar 7;22:101. doi: 10.1186/s12870-022-03488-x (PMC8900453; doi:10.1186/s12870-022-03488-x)
Supplement: Supplementary file 1 — Additional file 1: Fig. S1. Analyses of RNA editing sites in twelve Peucedanum plastomes: (A) numbers of RNA editing sites distributed in different codon positions; (B) numbers of RNA editing sites presented in genes. [file 12870_2022_3488_MOESM1_ESM.pdf]

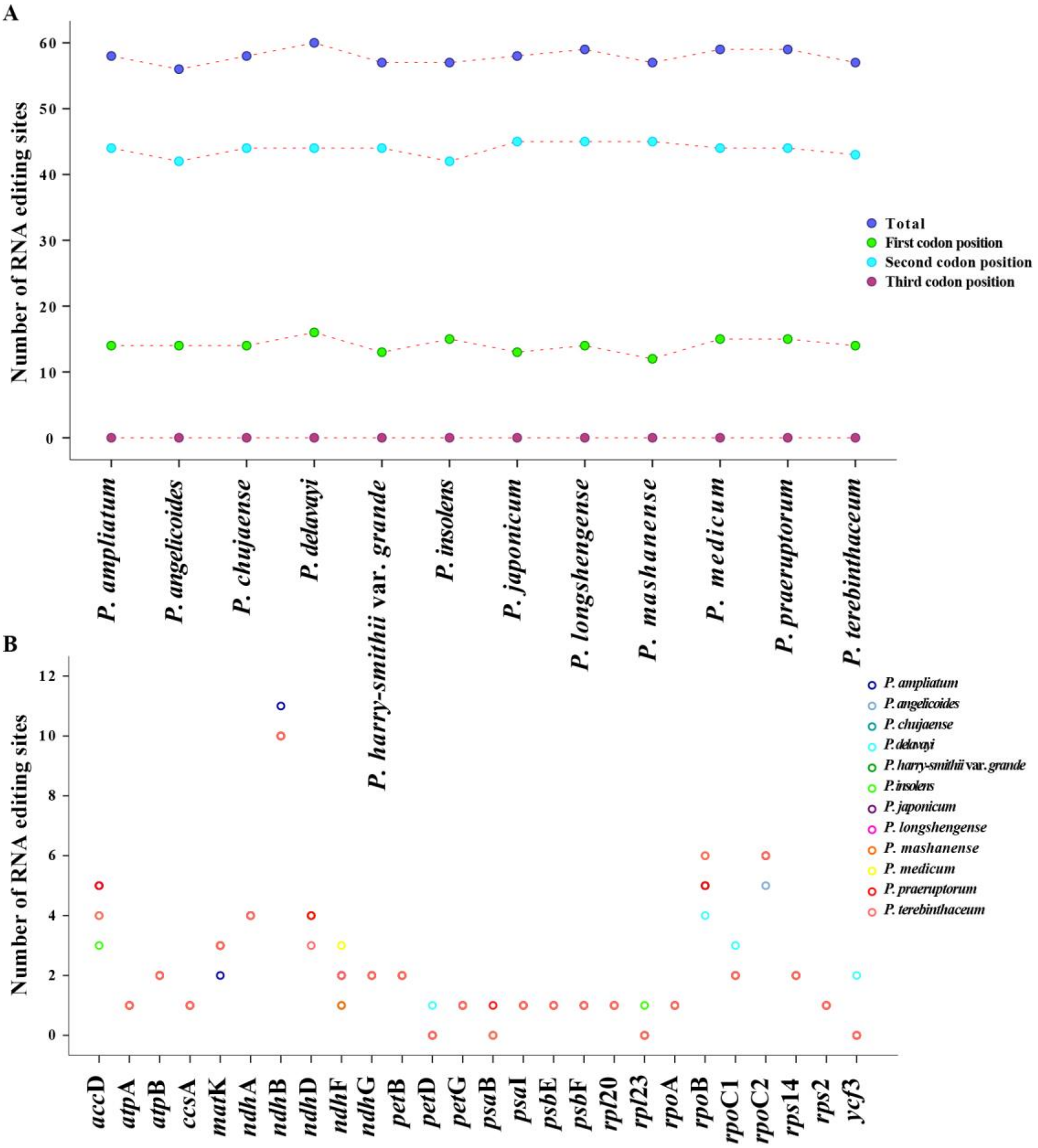

Fig. S1 Analyses of RNA editing sites in twelve *Peucedanum* plastomes: (A) numbers of RNA editing sites distributed in different codon positions; (B) numbers of RNA editing sites presented in genes.
