# Supplementary material for: The complete plastomes of seven Peucedanum plants: comparative and phylogenetic analyses for the Peucedanum genus
Source: BMC Plant Biol. 2022 Mar 7;22:101. doi: 10.1186/s12870-022-03488-x (PMC8900453; doi:10.1186/s12870-022-03488-x)
Supplement: Supplementary file 3 — Additional file 3: Table S2. List of unique genes identified in plastomes of Peucedanum. [file 12870_2022_3488_MOESM3_ESM.doc]

***Table S2 List of unique genes identified in plastomes of Peucedanum***.

| **Category of Genes** | **Group of gene** | **Name of gene** |
| --- | --- | --- |
| Self-replication | Ribosomal RNA genes | *rrn*4.5*,* *rrn*5*,* *rrn*16*, rrn*23 |
|  | Transfer RNA genes | *trn*C-GCA*, trn*D-GUC*, trn*E-UUC*, trn*F-GAA*, trn*G-GCC*, trn*G-UCC**, trn*H-GUG*, trn*I-CAU*, trn*K-UUU**, trn*L-CAA*, trn*L-UAA**, trn*L-UAG*, trn*M-CAU*, trn*P-UGG*, trn*Q-UUG*, trn*R-UCU*, trn*S-GCU*, trn*S-GGA*, trn*S-UGA*, trn*T-UGU*, trn*T-GGU^a^*, trn*V-GAC*, trn*V-UAC**, trn*Y-GUA*, trn*W-CCA*, trnf*M-CAU*, trn*A-UGC**, trn*I-GAU**, trn*N-GUU*,* *trn*R-ACG |
|  | Ribosomal protein (small subunit) | *rps*2*, rps*3*, rps*4*, rps*7*, rps*8*, rps*11*, rps*12***, rps*14*, rps*15*, rps*16**, rps*18*, rps*19 |
|  | Ribosomal protein (large subunit) | *rpl*2**, rpl*14*, rpl*16**, rpl*20*, rpl*22*, rpl*23*, rpl*32*, rpl*33*, rpl*36 |
|  | RNA polymerase | *rpo*A*, rpo*B*, rpo*C1**, rpo*C2 |
|  | Translational initiation factor | *inf*A |
| Genes for photosynthesis | Subunits of photosystem I | *psa*A*, psa*B*, psa*C*, psa*I*，psa*J*, ycf*3***, ycf*4 |
|  | Subunits of photosystem II | *psb*A*, psb*B*, psb*C*, psb*D*, psb*E*, psb*F*, psb*H*, psb*I*, psb*J*, psb*K*, psb*L*, psb*M*, psb*N*, psb*T*, psb*Z |
|  | Subunits of cytochrome | *pet*A*, pet*B**, pet*D**, pet*G*, pet*L*, pet*N |
|  | Subunits of ATP synthase | *atp*A*, atp*B*, atp*E*, atp*F**, atp*H*, atp*I |
|  | Large subunit of Rubisco | *rbc*L |
|  | Subunits of NADH dehydrogenase | *ndh*A**, ndh*B*, *, ndh*C*, ndh*D*, ndh*E*, ndh*F*, ndh*G*, ndh*H*, ndh*I*, ndh*J*, ndh*K |
| Other genes | Maturase | *mat*K |
|  | Envelope membrane protein | *cem*A |
|  | Subunit of acetyl-CoA | *acc*D |
|  | Synthesis gene | *ccs*A |
|  | ATP-dependent protease | *clp*P**** |
|  | Component of TIC complex | *ycf*1 |
| Genes of unknown function | Conserved open reading frames | *ycf*2*, ycf*15^b^ |

*: Gene with one intron

**: Gene with two introns.

a: Gene loss in *P. praeruptorum* and *P. harry-smithii* var. *grande*

b: Gene loss in *P. delavayi* and *P. insolens*
